# The economic burden of brucellosis in Western Iran

**DOI:** 10.1186/s41182-025-00860-z

**Published:** 2025-11-27

**Authors:** Meysam Behzadifar, Banafsheh Darvishi Teli, Samad Azari, Ahad Bakhtiari, Mariano Martini, Masoud Behzadifar

**Affiliations:** 1 Social Determinants of Health Research Center, Lorestan University of Medical Sciences, Khorramabad, Iran; 2 Health Management and Economics Research Center, School of Health Management and Information Sciences, Iran University of Medical Sciences, Tehran, Iran; 3 Health Management and Economics Research Center, Health Management Research Institute, Iran University of Medical Sciences, Tehran, Iran; 4 Health Equity Research Center (HERC), Tehran University of Medical Sciences (TUMS), Tehran, Iran; 5 Department of Health Sciences, University of Genoa, Genoa, Italy

**Keywords:** Brucellosis, Health expenditures, Cost of illness, Iran, Health policy

## Abstract

**Background:**

Brucellosis, a zoonotic disease caused by *Brucella* species, remains a major public health and economic challenge in regions dependent on livestock farming. In Iran, particularly in the western provinces, the disease imposes a heavy burden on healthcare systems and households through medical costs and productivity losses. This study quantified the economic burden of brucellosis in western Iran to inform policy and resource allocation.

**Methods:**

A cross-sectional cost-of-illness study was conducted among 427 brucellosis patients in Lorestan province**.** Data on direct medical costs (hospitalization, medications, diagnostics), direct non-medical costs (transportation, accommodation), and indirect costs (productivity losses) were collected using a bottom-up approach. All costs were expressed in 2024 USD, and sensitivity analyses were conducted at 0% and 5% discount rates. The catastrophic health expenditure (CHE) index was used to assess financial hardship.

**Results:**

The mean total cost per patient was 1,060 USD, with direct medical costs representing 73.6% of the total. Hospitalization was the largest component (38%), followed by surgical interventions (26%) and medications (13%). Indirect costs accounted for 18.9% of the total burden. The CHE index reached 66.3%, indicating severe financial strain for affected households. Sensitivity analyses showed notable variability in medical and non-medical cost estimates.

**Conclusion:**

Brucellosis imposes a substantial economic burden in western Iran, reflecting both healthcare and productivity losses. Effective control measures such as livestock vaccination, improved diagnosis, and expanded insurance coverage are essential to reduce financial hardship and support sustainable disease management.

**Supplementary Information:**

The online version contains supplementary material available at 10.1186/s41182-025-00860-z.

## Introduction

Brucellosis, a zoonotic disease caused by bacteria of the genus *Brucella*, remains a significant public health concern worldwide, particularly in regions where livestock farming is a cornerstone of the economy [1]. In Iran, brucellosis is endemic, with western regions reporting some of the highest incidence rates [2]. The disease not only poses severe health risks to humans, including chronic debilitating symptoms such as fever, joint pain, and fatigue, but also has far-reaching economic implications [3]. The loss of productivity due to illness, the cost of medical treatment, and the impact on livestock production collectively place a heavy burden on affected communities [4]. Despite being a preventable disease, brucellosis continues to challenge healthcare systems, particularly in resource-limited settings, due to inadequate surveillance, limited public awareness, and insufficient control measures in animal reservoirs [5].

The economic burden of brucellosis extends beyond direct healthcare costs, significantly affecting food security and livelihoods [6]. In western Iran, where agriculture and livestock farming are vital to the local economy, outbreaks of brucellosis can lead to substantial losses in livestock productivity, including reduced milk yield, abortions, and decreased fertility in animals [7]. These losses directly impact the income of farmers and the availability of animal-derived food products, exacerbating food insecurity in vulnerable populations [8]. Furthermore, the financial strain on the healthcare system, coupled with the need for prolonged treatment regimens, underscores the necessity of understanding the full economic impact of the disease [9]. Addressing these challenges requires a comprehensive assessment of the economic burden, which can inform targeted interventions and resource allocation [10].

Previous studies in other countries have examined the economic impact of brucellosis. For instance, research conducted in China revealed that brucellosis imposes a heavy financial burden on patients, especially among individuals with lower socioeconomic status, due to high out-of-pocket (OOP) expenses despite partial medical insurance coverage [11]. In India, an economic modeling study estimated substantial annual losses and disability-adjusted life years (DALYs) attributable to human brucellosis, highlighting its considerable health and economic burden [12]. Similarly, in Israel, a case–control study found that brucellosis significantly increased healthcare utilization costs, primarily due to elevated hospitalization and diagnostic expenditures [13]. In Armenia, a one-health-based evaluation demonstrated that existing test-and-slaughter control strategies entailed considerable public health and agricultural costs, emphasizing the importance of cross-sectoral collaboration for cost-effective control [14].

To date, however, no comprehensive studies have quantified the economic burden of brucellosis in Iran. This gap limits the ability of policymakers to design evidence-based interventions tailored to local contexts and economic realities. Therefore, this study aims to quantify the economic burden of brucellosis in western Iran, filling a critical knowledge gap in understanding the disease’s impact on both the healthcare system and the agricultural sector. By evaluating direct medical costs, indirect costs related to lost productivity, and the broader economic consequences for livestock production, this research provides valuable insights for policymakers and health system managers. The findings are expected to guide the development of cost-effective prevention and control strategies, ultimately reducing the disease’s burden on public health and the economy. For the Iranian health system, this study offers an evidence-based foundation for prioritizing brucellosis control programs, enhancing food security, and improving the overall well-being of affected communities.

## Methods

### Design and population

This study utilized a cross-sectional design to conduct a partial economic evaluation and cost-of-illness (COI) analysis for brucellosis in western Iran [15]. The study population included all brucellosis patients in Lorestan province, western Iran. The sampling framework was based on the brucellosis patient registry maintained by the Health Department of Lorestan University of Medical Sciences, which compiles data from public hospitals and primary healthcare centers in the region. Private hospitals were not included due to the limited reporting of confirmed brucellosis cases.

Eligible participants were individuals residing in Lorestan province until the study’s conclusion, who provided informed consent, and who received continuous treatment either as outpatients or inpatients. Inclusion criteria were: (1) confirmed diagnosis of brucellosis based on clinical and laboratory criteria (positive Wright, 2ME, or ELISA tests), (2) residence in Lorestan province, and (3) willingness to participate. Exclusion criteria were: (1) incomplete medical or cost data and (2) refusal to participate. To ensure a representative sample, patients were recruited from diverse demographic backgrounds and disease stages. Both hospital-treated and primary care–managed patients were included. Stratified sampling with proportional allocation was employed to include patients from both urban and rural areas.

The sample size was calculated using the following formula:

$${\text{n}}\, = \,{{\left( {{\text{Z}}^{2} \, \times \,\sigma^{2} } \right)} \mathord{\left/ {\vphantom {{\left( {{\text{Z}}^{2} \, \times \,\sigma^{2} } \right)} {{\text{d}}^{2} }}} \right. \kern-0pt} {{\text{d}}^{2} }}$$

where *Z* = 1.96 (for 95% confidence interval), *σ* = standard deviation of total cost from a pilot study (US$ 950), and *d* = desired precision (US$ 100). Based on this calculation, a minimum of 345 patients was required; however, 427 patients were included to enhance statistical power and representativeness.

**Fig. 1:**
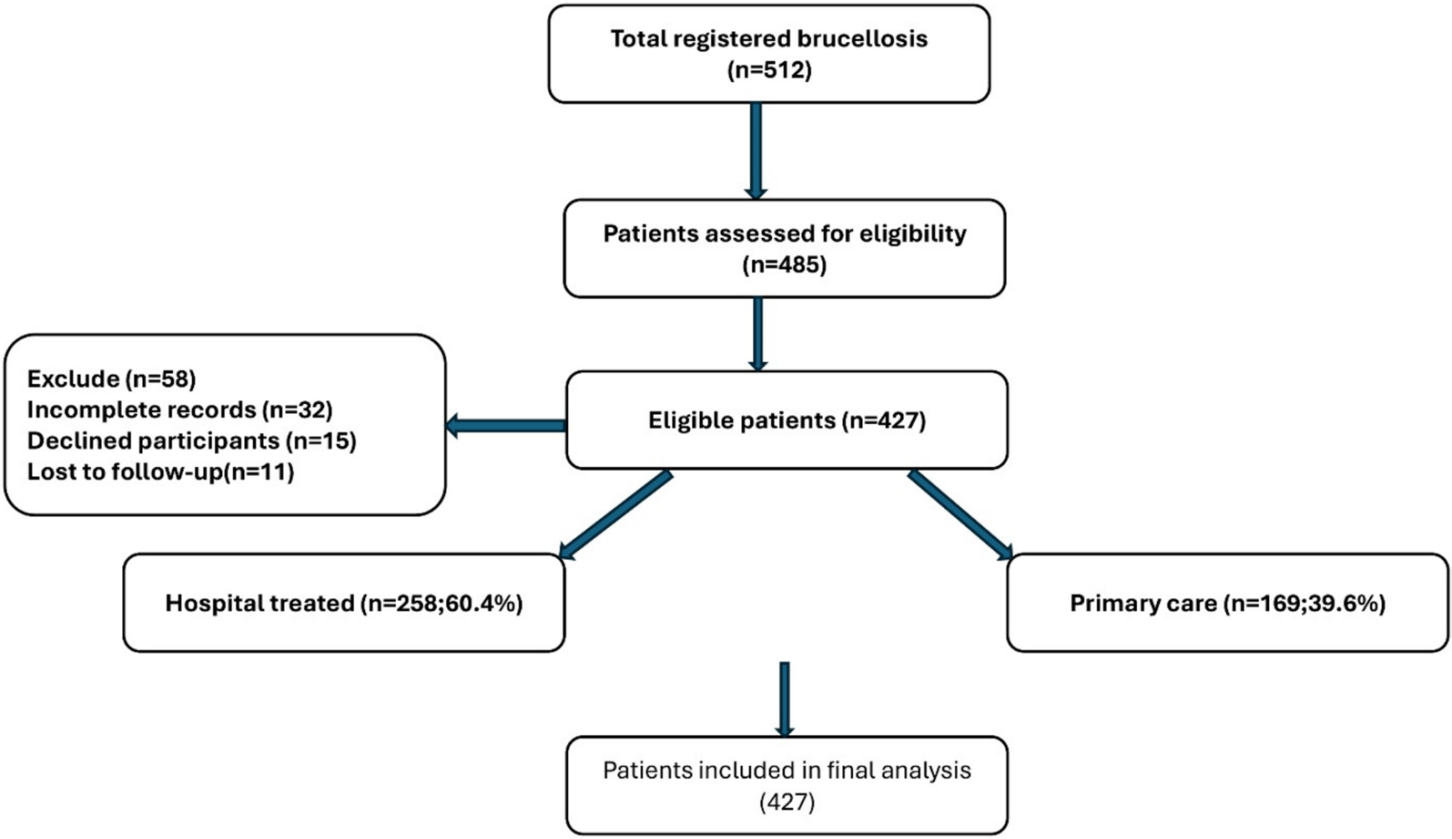
Flowchart of patient selection and inclusion process for brucellosis cases in Lorestan Province, Iran

The process of patient selection and inclusion is summarized in Fig. 1. A total of 512 registered brucellosis cases were identified from the Lorestan Health Department registry between November 2023 and November 2024. After eligibility assessment, 58 patients were excluded due to incomplete medical records (n = 32), refusal to participate (n = 15), or loss to follow-up (n = 11). The remaining 427 patients met all inclusion criteria and were enrolled in the study. Among these, 60.4% (n = 258) were hospital-treated (inpatient) cases and 39.6% (n = 169) were managed in primary care or outpatient settings. This sampling approach ensured that both mild and severe cases were represented, enhancing the representativeness of the study population for western Iran.

A bottom-up approach was adopted for cost calculation, considering a societal perspective, and a prevalence-based analysis was conducted to estimate the costs of brucellosis during the study period. The economic evaluation covered all cases diagnosed and treated between November 2023 and November 2024, representing a one-year observation window for data collection and cost estimation. All costs for 2024 were initially reported in Iranian Rials using the official average exchange rate provided by the World Bank (1 USD = 42,000 Rials) [16]. To enhance cross-country comparability, costs were also converted using the Purchasing Power Parity (PPP) exchange rate for 2024.

### Data collection

Data were collected using a structured form developed specifically for this study in consultation with infectious disease specialists and health economists. The form included sections on demographic information, direct medical costs, direct non-medical costs, indirect costs, and out-of-pocket expenses. Demographic data, such as age, marital status, education level, insurance coverage, and disease stage, were obtained from patient medical records and telephone interviews with patients or their caregivers. All medical records were sourced from public hospitals affiliated with Lorestan University of Medical Sciences. The questionnaire used in this study has not been published elsewhere and was developed by the authors for this specific research. An English version of the questionnaire is provided as a supplementary file (Supplementary File 1) and is cited in the main manuscript.

### Costs

**Direct Medical Costs (DMC):** These included expenses related to diagnostic procedures (e.g., serological tests such as Rose Bengal, ELISA, Wright test, blood cultures, and PCR tests), medications (e.g., antibiotics like doxycycline, rifampin, and streptomycin, as well as supportive therapies), hospitalization (inpatient care for severe cases, including room charges, nursing care, and ICU stays if necessary), outpatient visits (consultations with general practitioners and infectious disease specialists, as well as follow-up appointments), imaging (X-rays, ultrasounds, or CT scans for complications such as osteomyelitis, abscesses, or joint damage), surgical interventions (e.g., abscess drainage or joint replacement), and rehabilitation services (e.g., physiotherapy for chronic joint or musculoskeletal issues). Costs were based on unit tariffs set by the Ministry of Health and Medical Education (MOHME), multiplied by the quantity of each service used per patient to obtain the total DMC.

**Direct Non-Medical Costs (DNMC):** These comprised transportation expenses, accommodation costs, meal expenses for patients and their companions, and caregiver-related costs (e.g., time off work for family members providing informal care). These figures were derived from interviews with patients and their relatives. Average market prices for transportation and accommodation in Lorestan during 2024 were used to estimate these unit costs.

**Indirect Costs:** These were calculated based on productivity losses due to work absenteeism and premature mortality among patients and caregivers. The human capital approach was used, applying the following formula:

$${\text{Indirect Cost}}\, = \,\left( {{\text{Number}}\,{\text{of}}\,{\text{workdays}}\,{\text{lost}}\, \times \,{\text{Average}}\,{\text{daily}}\,{\text{wage}}} \right)\, + \,\left( {{\text{Years}}\,{\text{of}}\,{\text{potential}}\,{\text{life}}\,{\text{lost}}\, \times \,{\text{Annual}}\,{\text{wage}}} \right)$$

The mean annual wage in Iran in 2024, according to the Iranian Statistics Center, was 2,160,000,000 Rials (equivalent to approximately USD 51,428 using the official exchange rate). Absenteeism costs were estimated by multiplying the number of workdays missed by the average daily wage. Although the human capital method may overestimate productivity losses in low-income settings, it was selected for consistency with comparable studies; this limitation is discussed in the manuscript.

### Calculation of the economic burden of brucellosis

The economic burden of brucellosis was calculated using the following formula [17], which incorporates the estimated mean direct and indirect costs per patient and the prevalence rate of brucellosis in the region:

$${\text{Economic}}\,{\text{Burden}}\, = \,{\text{Total}}\,{\text{Cost}}\,\left( {{\text{Direct}}\,{\text{Medical}}\,{\text{Cost}}\, + \,{\text{Direct}}\,{\text{Non - Medical}}\,{\text{Cost}}\, + \,{\text{Indirect}}\,{\text{Cost}}} \right)\, \times \,{\text{Number}}\,{\text{of}}\,{\text{Brucellosis}}\,{\text{Patients}}$$

This study reports annual costs for 2024 and includes 427 brucellosis patients in Lorestan province. A probabilistic sensitivity analysis (PSA) and multivariate scenario testing were also conducted to capture uncertainty in cost estimates. Sensitivity analyses were conducted using varying exchange rate scenarios to assess robustness.

### Sensitivity analysis

A sensitivity analysis was performed to evaluate the impact of varying economic conditions and assumptions on the estimated economic burden of brucellosis. Two discount rates were applied:

I. • 0% discount rate: This scenario assumes no discounting over time, treating future costs as equivalent to present costs. It provides a baseline estimate of the immediate economic burden.
II. • 5% discount rate: This rate reflects the time value of money, discounting future costs to their present value. It is commonly used in health economic evaluations to account for the opportunity cost of capital.

A tornado diagram was created to illustrate the relative influence of key cost components, such as hospitalization and productivity losses, on total economic burden. The directionality of each variable is indicated by the length and orientation of the bars, representing sensitivity to parameter variation.

By comparing these scenarios, the study aimed to capture a range of potential outcomes and enhance the reliability of the economic burden estimates.

### Catastrophic Health Expenditure (CHE) index calculation

To assess the financial impact of brucellosis on households, the Catastrophic Health Expenditure (CHE) index was calculated. CHE was defined as the proportion of households whose out-of-pocket (OOP) healthcare expenses exceeded 40% of their capacity to pay (CTP). The 40% threshold follows WHO recommendations; however, alternative thresholds (10% and 25%) were also tested for sensitivity analysis. The capacity to pay was estimated as the household’s annual income after accounting for basic subsistence needs. Annual household income was estimated through patient interviews and cross-validated with data from the Iranian Household Expenditure and Income Survey (2024).

$${\text{CHE}}\,{\text{Indicator}}\, = \,\left( {{{\text{OOP Costs}} \mathord{\left/ {\vphantom {{\text{OOP Costs}} {{\text{CTP}}}}} \right. \kern-0pt} {{\text{CTP}}}}} \right)\, \times \,{1}00$$

Although CHE is not a direct measure of economic burden, it provides a complementary perspective on household financial vulnerability due to brucellosis-related costs.

### Statistical analysis

Data were analyzed using R software (Version 4.4.1). Descriptive statistics were used to summarize demographic characteristics and cost data. Sensitivity and probabilistic analyses were conducted to evaluate the robustness of the findings under different economic assumptions and discount rates. Capital costs were excluded because no major fixed investments were made during the study period. Societal costs, such as productivity losses of unpaid household workers, were considered under the indirect cost category.

This methodological framework provides a comprehensive approach to estimating the economic burden of brucellosis in western Iran, ensuring that both direct and indirect costs are captured and the financial impact on households is thoroughly assessed.

### Ethical considerations

This study was reviewed and approved by the Ethics Committee of Lorestan University of Medical Sciences (Approval Code: IR.LUMS.REC.1403.336). All participants were informed about the study objectives, and written informed consent was obtained before data collection. Participation was voluntary, and confidentiality of all personal information was strictly maintained. All methods were performed in accordance with relevant guidelines and regulations. No additional institutional or governmental permissions were required for conducting this study.

## Results

### Demographic characteristics

**Fig. 2:**
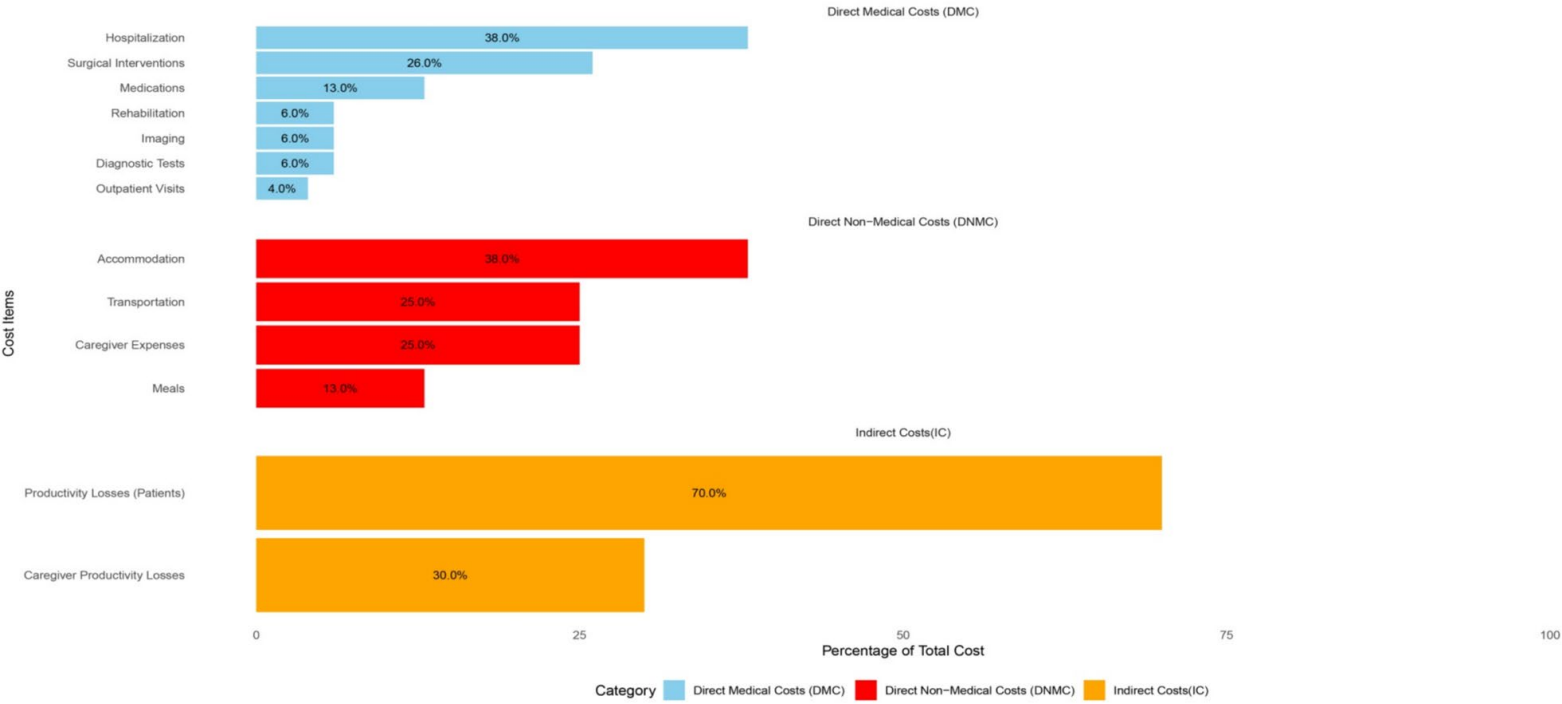
The costs and percentages for items related to DMC, DNMC, and indirect costs are detailed

Table 1 outlines the demographic profile of the study participants. The age distribution reveals that the largest proportion of participants (30%) were aged 25–34 years, followed by those aged 35–44 years (25%). Younger participants (15–24 years) accounted for 20%, while older age groups (45–54 years, 55–64 years, and > 65 years) represented 15%, 7.5%, and 2.5% of the sample, respectively. The majority of participants were married (70%), with smaller proportions being single (20%), divorced (5%), or widowed (5%). Employment status was nearly evenly split, with 60% employed and 40% unemployed. Geographically, 60% of participants resided in urban areas, while 40% lived in rural settings. Education levels were distributed equally among under diploma, diploma, and higher than diploma categories (30% each), with 10% being illiterate. Insurance coverage was primarily through rural insurance (40%) and social security (30%), while 10% had no insurance. Additionally, 60% of participants reported having supplemental insurance. Standard deviations and 95% confidence intervals were calculated for continuous demographic variables (e.g., age, income), as shown in Table 1. Figure 2 shows the different costs for patients with brucellosis.

**Table 1:** Demographic characteristics of patients

Variable	Category	Number	Percent	Mean ± SD/95% CI
Age (years)	15–24	85	20.0	**Mean = 33.8 ± 12.6 (95% CI 32.0–35.6)**
	25–34	128	30.0	
	35–44	107	25.0	
	45–54	64	15.0	
	55–64	32	7.5	
	> 65	11	2.5	
Employment status	Employed	256	60.0	
	Unemployed	171	40.0	
Marital status	Married	299	70.0	
	Single	85	20.0	
	Divorced	21	5.0	
	Widowed	22	5.0	
Residential location	Urban	256	60.0	
	Rural	171	40.0	
Education status	Illiterate	43	10.0	
	Under diploma	128	30.0	
	Diploma	128	30.0	
	Higher than diploma	128	30.0	
Insurance status	Rural insurance	171	40.0	
	Social Security	128	30.0	
	Armed Forces	43	10.0	
	Iran Health	43	10.0	
	No insurance	42	10.0	
Supplemental insurance	Yes	256	60.0	
	No	171	40.0	
Annual household income (USD)		–	–	**Mean = 4,000 ± 950 (95% CI 3.840–4.160)**

Bold values indicate subtotal or aggregated category-level costs for readability

### Economic burden of brucellosis in Western Iran

**Fig. 3:**
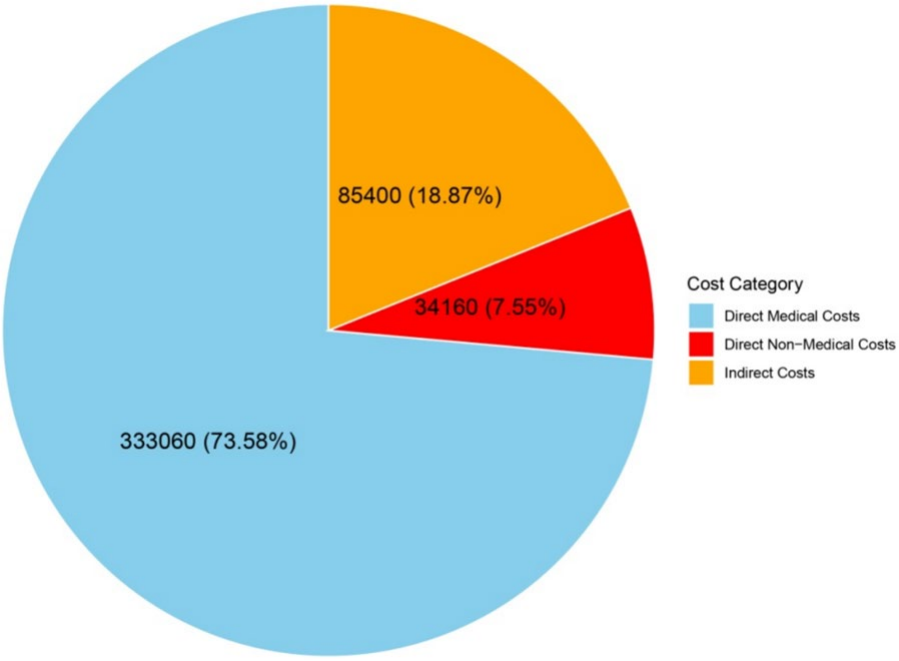
The economic burden of Brucellosis is composed of direct non-medical costs and indirect costs

Table 2 outlines the economic burden of brucellosis in western Iran, categorized into DMC, DNMC, and indirect costs. DMC accounted for the largest share of total costs (73.58%), with hospitalization being the most significant expense (38% of DMC). Surgical interventions and medications also contributed substantially, representing 26% and 13% of DMC, respectively. Diagnostic tests, imaging, and rehabilitation each accounted for 6% of DMC, while outpatient visits made up 4%. DNMC constituted 7.55% of total costs, with accommodation (38% of DNMC) and transportation (25% of DNMC) being the primary contributors. Meals and caregiver expenses each represented 13% and 25% of DNMC, respectively. Indirect costs, representing 18.87% of total costs, were primarily driven by productivity losses among patients (70% of indirect costs) and their caregivers (30%). The total mean cost per patient was 1,060 USD (SD: ± 180 USD; 95% CI: 890–1,230 USD). Figure 2 represents the total costs across all three categories. The economic burden is broken down by cost category (direct medical, direct non-medical, and indirect) in Fig. 3.

**Table 2:** Analysis of healthcare costs for brucellosis in Lorestan province, Western Iran: Direct medical, direct non-medical, and indirect costs with mean and percentage distribution

Type of cost	Item	Mean cost per patient (USD)	Total cost (USD)	Percent within category	Percent of total	SD ( ±)	95% CI
Direct Medical Costs (DMC)	Diagnostic tests	50	21,350	6	4.6	± 12	47–53
	Medications	100	42,700	13	9.4	± 25	95–105
	Hospitalization	300	128,100	38	28.3	± 60	290–310
	Outpatient visits	30	12,810	4	2.8	± 8	28–32
	Imaging	50	21,350	6	4.6	± 10	48–52
	Surgical interventions	200	85,400	26	18.9	± 40	190–210
	Rehabilitation	50	21,350	6	4.6	± 10	48–52
Subtotal DMC	–	**780 ± 110**	**333,060**	100	**73.58**	–	95% CI 750–810
Direct Non-Medical Costs (DNMC)	Transportation	20	8,540	25	1.9	± 5	18–22
	Accommodation	30	12,810	38	2.8	± 7	28–32
	Meals	10	4,270	13	0.9	± 3	9–11
	Caregiver expenses	20	8,540	25	1.9	± 5	18–22
Subtotal DNMC	–	**80 ± 15**	**34,160**	100	**7.55**	–	95% CI 75–85
Indirect Costs	Productivity loss (patients)	150	64,050	70	14.2	± 30	145–155
	Productivity loss (caregivers)	50	21,350	30	4.7	± 10	48–52
Subtotal Indirect Costs	–	**200 ± 35**	**85,400**	100	**18.87**	–	95% CI 190–210
Total Cost per Patient	–	**1,060 ± 180**	**452,620**	100	100	–	95% CI 890–1,230

Bold values indicate subtotal or aggregated category-level costs for readability

Based on the annual incidence of brucellosis in Lorestan province (estimated at 40 cases per 100,000 population) and a provincial population of approximately 2 million, the total annual economic burden was estimated at around 848,000 USD (95% CI: 720,000–980,000 USD). Extrapolating this burden to the national level, assuming similar incidence and cost patterns, suggests an estimated total annual economic impact of approximately 35–40 million USD across Iran (95% CI: 29–46 million USD). These provincial and national projections provide a broader understanding of the financial impact of brucellosis beyond individual-level costs and can inform regional and national policy planning.

### Catastrophic health expenditure (CHE) index

The OOP cost for brucellosis treatment was calculated at 1,060 USD per patient, while the mean annual household income was 4,000 USD. The capacity to pay (CTP), defined as 40% of household income after accounting for basic needs, was estimated at 1,600 USD annually. Using these values, the CHE indicator was calculated as follows:

$${\text{CHE}}\,{\text{Indicator}}\,{ = }\,\left( { \frac{{\text{Annual OOP cost}}}{{{\text{CTP}}}}} \right)\, \times \,100\, = \,\frac{1060}{{1600}}\, \times \,100\, = \,66.25\%$$

(All monetary values were converted to USD using the official World Bank average exchange rate for 2023–2024, equal to 1 USD = 42,000 Rials).

This result demonstrates that the CHE indicator exceeds the critical threshold of 40%, indicating that a significant proportion of households in the cohort face CHE under these conditions. The high CHE value highlights the substantial financial burden imposed by brucellosis, which may lead to impoverishment or severe economic strain for affected households.

This finding further supports the provincial and national estimates, suggesting that the economic impact of brucellosis poses a substantial financial risk to households and health systems alike.

### Sensitivity analysis

#### Sensitivity analysis using tornado diagram

**Fig. 4:**
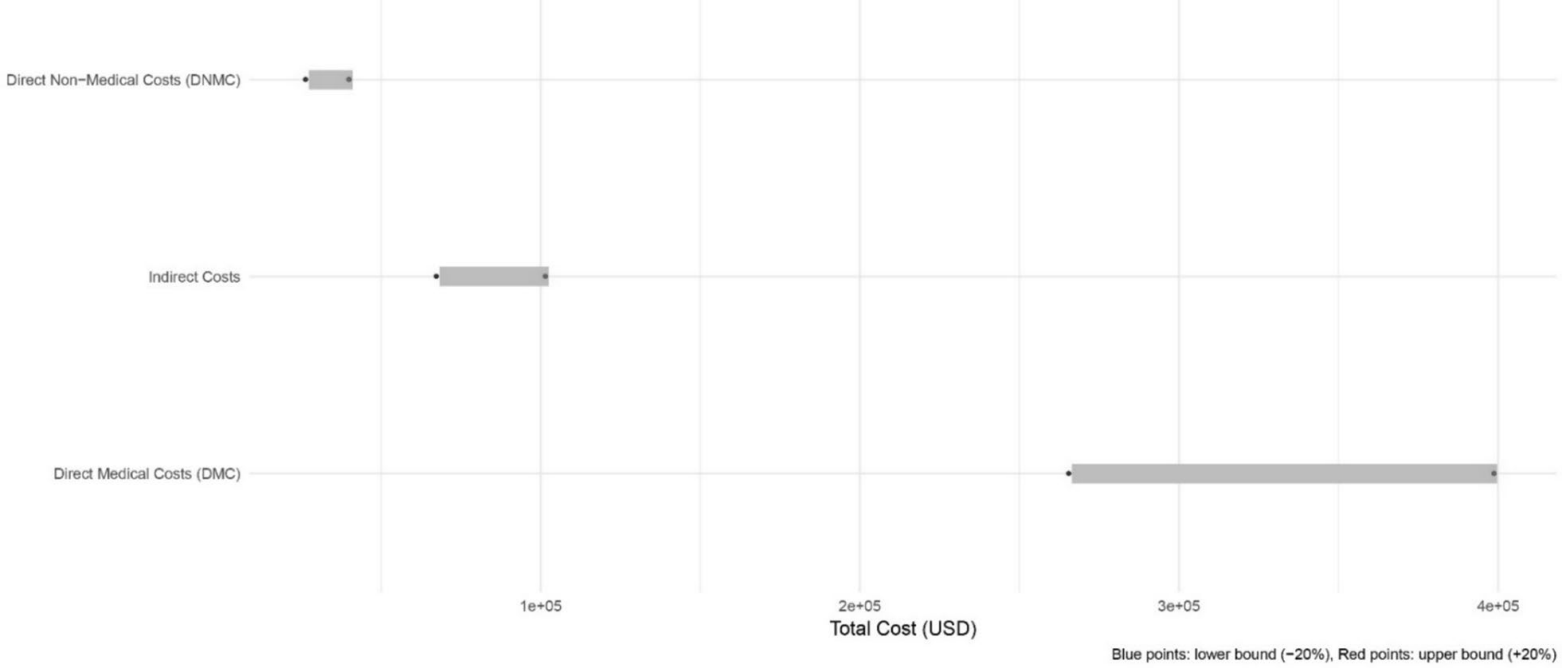
illustrates the results of the one-way sensitivity analysis for the economic burden of brucellosis. The tornado diagram displays how variations of ± 20% in each cost category DMC, DNMC, and indirect costs affect the total estimated cost per patient. The horizontal bars represent the range of variation in total costs associated with each cost component, with blue points indicating the lower bound (− 20%) and red points indicating the upper bound (+ 20%) of the sensitivity range. The wider the bar, the greater the impact of uncertainty in that parameter on the overall economic burden. As shown, DMC exert the greatest influence on total costs, followed by indirect costs and DNMC. This pattern indicates that medical expenses, particularly hospitalizations and surgical procedures, are the key drivers of the overall economic burden of brucellosis in western Iran. The diagram demonstrates that the model’s results are most sensitive to changes in DMC, highlighting the importance of improving accuracy in estimating medical expenditures when conducting economic burden assessments. The relatively smaller ranges for DNMC and indirect costs suggest that these components have less uncertainty or lower variability in total cost estimates

To evaluate the robustness of cost estimates and identify key cost drivers, a one-way sensitivity analysis was performed. The Tornado diagram was used to illustrate how variations in individual cost components (DMC, DNMC, and Indirect Costs) affect the total estimated economic burden of brucellosis (Fig. 4). Each cost component was varied by ± 20% from its baseline value while keeping the others constant, and the resulting changes in total cost were plotted as horizontal bars. The length of each bar in the Tornado diagram represents the degree of influence of that cost component on the total cost estimate, with longer bars indicating greater sensitivity. This visualization helps identify which parameters have the strongest impact on model uncertainty, thereby guiding future data collection and policy focus.

#### Probabilistic sensitivity analysis (Monte Carlo simulation)

**Fig. 5:**
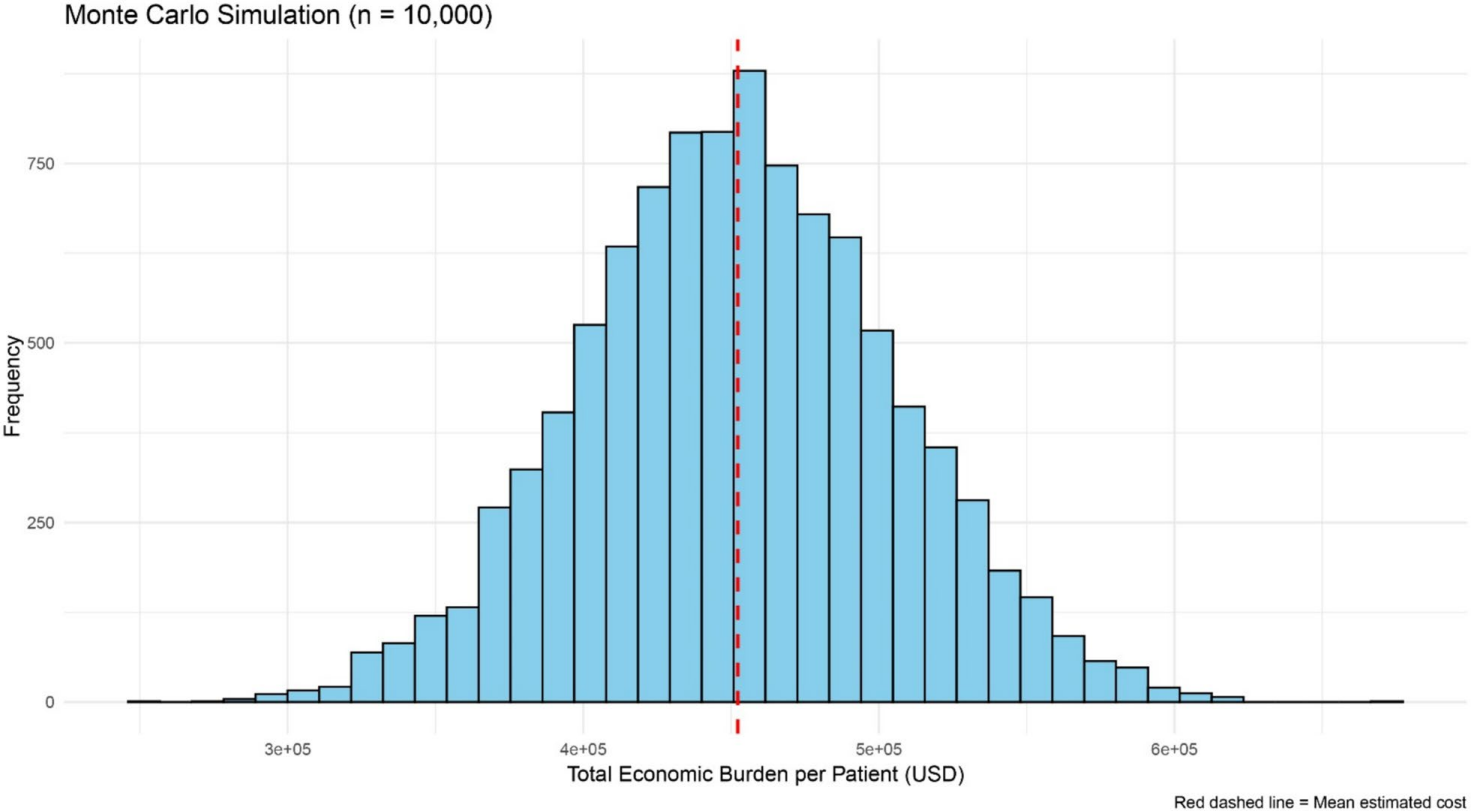
presents the results of the Monte Carlo simulation (n = 10,000 iterations) used to quantify the uncertainty surrounding the total estimated economic burden per brucellosis patient. In this analysis, direct medical, non-medical, and indirect costs were randomly varied within their specified distributions (assuming normality) to simulate the potential range of total costs. The histogram illustrates the distribution of simulated total costs, while the red dashed vertical line represents the mean estimated total cost obtained across all iterations. The majority of simulated outcomes cluster around the mean, indicating a relatively stable central estimate, though some spread exists due to input uncertainty. The simulation results confirm that the mean total economic burden per patient is approximately 1,060 USD, with a 95% confidence interval derived from the simulations reflecting the uncertainty in model parameters. This probabilistic analysis complements the deterministic tornado diagram by incorporating joint parameter uncertainty, thereby strengthening the robustness of the study’s cost estimates and providing a more realistic depiction of the potential range of economic outcomes associated with brucellosis

To further account for uncertainty in cost estimates, a Monte Carlo simulation with 10,000 iterations was conducted (Fig. 5). Each cost component (DMC, DNMC, and Indirect Costs) was assigned a normal probability distribution defined by its mean and standard deviation. Random sampling was performed iteratively to generate distributions of total costs across simulations. This probabilistic approach provides a range of likely economic outcomes rather than a single deterministic estimate**,** allowing the estimation of mean, standard deviation, median, and 95% confidence intervals for the total economic burden. The results are presented as both histograms and cumulative probability plots.

## Discussion

The findings of this study provide a comprehensive assessment of the economic burden of brucellosis in western Iran, highlighting its significant impact on both healthcare systems and households. The demographic profile of the study participants reflects the widespread nature of the disease, particularly among individuals aged 25–44 years, who constitute the majority of the affected population. This age group, often representing the most economically active segment of society, underscores the substantial productivity losses associated with brucellosis. The high proportion of married participants (70%) further emphasizes the disease's ripple effect on families, as caregivers and household incomes are also adversely affected. The geographic distribution of cases, with 40% of participants residing in rural areas, aligns with the endemic nature of brucellosis in regions where livestock farming is a primary livelihood. This highlights the critical link between zoonotic disease control and agricultural sustainability in resource-limited settings [18].

The economic burden of brucellosis, as quantified in this study, is substantial, with DMC accounting for the largest share (73.58%) of total costs. Hospitalization emerged as the most significant contributor to DMC, reflecting the severe health complications associated with advanced or untreated brucellosis [19]. Surgical interventions and medications also represented considerable expenses, underscoring the need for early diagnosis and effective treatment to mitigate these costs [20]. DNMC, though smaller in proportion, was driven primarily by accommodation and transportation expenses, which disproportionately affect rural populations with limited access to healthcare facilities. Indirect costs, representing 18.87% of the total burden, were largely attributed to productivity losses among patients and their caregivers, further exacerbating the economic strain on households and communities.

Capital costs, such as medical equipment, hospital infrastructure depreciation, and facility maintenance, were not included in this analysis due to the focus on patient-level financial impacts rather than institutional expenditures. Similarly, broader societal costs such as long-term productivity losses beyond the study period or the economic effects on livestock markets were excluded to maintain consistency with the cost-of-illness framework and ensure comparability with previous studies [11, 12].

It should also be noted that the study applied the human capital approach to estimate productivity losses. Although widely used in economic burden analyses, this method may overestimate indirect costs in low-income or informal labor settings because it assumes full employment and continuous productivity. Future studies could complement these findings by applying the friction-cost method, which provides a more conservative estimate by considering the time required to replace a sick worker rather than the entire period of absence.

The CHE index of 66.25% reveals the profound financial burden imposed by brucellosis on affected households. With OOP costs exceeding 40% of the CTP for a significant proportion of families, the risk of impoverishment and economic hardship is alarmingly high. The 40% threshold used in this study aligns with WHO’s definition of catastrophic health expenditure; however, sensitivity analyses using alternative thresholds (10% and 25%) also confirmed that a large share of households still faced catastrophic spending. This finding is particularly concerning in a region where agriculture and livestock farming are vital to the local economy, as the financial strain from brucellosis can perpetuate cycles of poverty and food insecurity [21]. The reported CHE prevalence (66.25%) is consistent with findings from studies on other chronic and infectious diseases in Iran, such as cancer and tuberculosis, where CHE levels have ranged between 50 and 70%, suggesting that the result is plausible within the national healthcare financing context. The high CHE index underscores the urgent need for policy interventions to reduce OOP expenditures, such as expanding insurance coverage and subsidizing treatment costs for vulnerable populations [22].

The sensitivity analysis further highlights the variability and uncertainty in estimating the economic burden of brucellosis. The wide ranges for DMC and DNMC, particularly under a 5% discount rate, emphasize the need for robust data collection and careful consideration of assumptions in economic evaluations. The application of a discount rate, while standard in health economic analyses, introduces additional uncertainty, particularly for indirect costs [23]. This variability underscores the importance of conducting sensitivity analyses to assess the robustness of findings and inform decision-making under different scenarios [24].

The results of this study align with previous research on the economic burden of brucellosis in other endemic regions, which have similarly identified high direct medical costs and significant productivity losses as key drivers of the disease's economic impact. For instance, a study by He et al. in Xinjiang, China, found that the median direct cost per brucellosis episode was USD 688.65, with out-of-pocket expenses amounting to USD 391.44, which exceeded both the regional and national per capita health expenditures [11]. This is consistent with our findings, where direct medical costs were the largest component of the total economic burden. The study also highlighted the inequity in economic consequences across different socioeconomic status (SES) groups, with lower SES patients spending a higher percentage of their income on healthcare (37.23%) compared to higher SES groups (12.96%). This mirrors our findings in western Iran, where the CHE index was particularly high, indicating that brucellosis disproportionately affects economically vulnerable populations.

Similarly, Singh et al. conducted a cost–benefit analysis of brucellosis control interventions in India and found that vaccination alone was highly cost-effective, with benefit–cost ratios ranging from 7.98 to 21.27 [25]. This study emphasized the importance of vaccination as a cost-effective strategy for reducing the economic burden of brucellosis, particularly in low-income settings. Our study also underscores the need for cost-effective interventions, such as vaccination, to mitigate the high direct and indirect costs associated with brucellosis. However, unlike the Indian study, which focused on the economic benefits of vaccination, our study highlights the immediate financial strain on households due to high OOP costs, suggesting that while vaccination is a long-term solution, immediate financial support mechanisms are also needed to alleviate the burden on affected families.

In another study, Kitza et al. conducted a systematic review of economic assessments for brucellosis control interventions in livestock populations across nine countries [26]. They found that vaccination alone was cost-effective in most cases, with BCRs ranging from 3.2 to 21.3, while test-and-slaughter interventions were generally not cost-effective. This aligns with our findings that emphasize the need for cost-effective control measures, particularly in regions where livestock farming is a primary livelihood. The review also highlighted the importance of sensitivity analyses in economic evaluations, which is consistent with our approach in assessing the variability and uncertainty in cost estimates [26].

However, the unique socio-economic context of western Iran, characterized by a heavy reliance on livestock farming and limited healthcare resources, amplifies the challenges associated with brucellosis control. The findings highlight the need for a multi-sectoral approach to address the disease, integrating public health interventions with agricultural and economic policies [27]. For example, while vaccination is a proven cost-effective strategy, as demonstrated in the studies by Singh et al. [25] and Kitza et al. [26], its implementation in western Iran may require additional support, such as subsidies for vaccines and improved access to veterinary services, particularly in rural areas. This study provides critical evidence on the economic burden of brucellosis in western Iran, emphasizing its far-reaching implications for healthcare systems, households, and the agricultural sector. The high direct and indirect costs, coupled with the substantial risk of catastrophic health expenditure, underscore the urgency of implementing cost-effective prevention and control strategies. Policymakers should prioritize measures such as improving diagnostic and treatment access, enhancing public awareness, and strengthening livestock vaccination programs to reduce the disease's burden. Future research should focus on refining cost estimates and evaluating the cost-effectiveness of interventions to ensure sustainable and equitable solutions for brucellosis control in endemic regions.

## Limitations

While this study provides valuable insights into the economic burden of brucellosis in western Iran, it is not without limitations. First, the reliance on self-reported data for some cost components, such as direct non-medical costs and out-of-pocket expenses, may introduce recall bias or inaccuracies. Second, possible recall bias, selection bias (from including primarily registered or hospitalized cases), and survivorship bias (due to exclusion of fatal cases) must be acknowledged, as these factors could lead to underestimation or overestimation of the true economic burden. Third, the study’s cross-sectional design limits the ability to capture the long-term economic impact of brucellosis, particularly for patients with chronic or recurrent symptoms. Fourth, the sampling framework, based on the brucellosis patient registry, may exclude individuals who did not seek medical care or were treated in private healthcare facilities, potentially underestimating the true burden of the disease. Fifth, the use of a single year’s exchange rate for cost calculations may limit comparability with studies from other years or regions, despite the application of sensitivity analyses to address this issue. Furthermore, currency fluctuations and PPP adjustments may affect international comparability, although using the World Bank’s average exchange rate (1 USD = 42,000 Rials) helped standardize cost reporting. Finally, the study’s focus on Lorestan province may limit the generalizability of findings to other regions of Iran or countries with different healthcare systems and economic contexts. Challenges in data collection included incomplete or inaccurate diagnostic records for some patients, as well as difficulties in accessing a subset of patients with comprehensive information. Additionally, some patients passed away before data collection could be completed. Future research should address these limitations by incorporating longitudinal data, expanding the geographic scope, and refining cost estimation methods.

## Conclusion

This study highlights the substantial economic burden of brucellosis in western Iran, driven by high direct medical costs, significant productivity losses, and the risk of catastrophic health expenditure for affected households. The findings underscore the need for targeted interventions to reduce the disease’s impact on public health and the economy. Policymakers should prioritize measures such as improving access to early diagnosis and treatment, expanding insurance coverage, and implementing livestock vaccination programs to control brucellosis at its source. Additionally, public awareness campaigns and educational initiatives can play a critical role in preventing transmission and reducing the disease’s burden. The sensitivity analysis emphasizes the importance of robust data collection and careful consideration of assumptions in economic evaluations, providing a foundation for future research to refine cost estimates and evaluate the cost-effectiveness of interventions. By addressing the economic and health challenges posed by brucellosis, Iran can enhance food security, protect livelihoods, and improve the overall well-being of its population. This study serves as a call to action for integrated, multi-sectoral efforts to combat brucellosis and mitigate its far-reaching consequences.

## Supplementary Information

Supplementary material 1. Structured questionnaire for assessing the economic burden of brucellosis in Western Iran.

## Data Availability

The datasets generated and/or analysed during the current study are not publicly available due but are available from the corresponding author on reasonable request.

## Abbreviations

COI: Cost-of-illness
TDMC: Direct medical costs
MOHME: Ministry of Health and Medical Education
DNMC: Direct non-medical costs
CHE: Catastrophic health expenditure
OOP: Out-of-pocket
CTP: Capacity to pay
SES: Socioeconomic status
DALYs: Disability-adjusted life years
CI: Confidence intervals
PSA: Probabilistic sensitivity analysis
