# Supplementary material for: The economic burden of brucellosis in Western Iran
Source: Trop Med Health. 2025 Nov 27;53:174. doi: 10.1186/s41182-025-00860-z (PMC12661891; doi:10.1186/s41182-025-00860-z)
Supplement: Supplementary file 1 — Supplementary material 1. Structured questionnaire for assessing the economic burden of brucellosis in Western Iran. [file 41182_2025_860_MOESM1_ESM.docx]

**Questionnaire for assessing the economic burden of brucellosis: direct and indirect costs, out-of-pocket expenses, and financial impact on households**

**Purpose of the Questionnaire:**

This questionnaire is designed to collect information about the economic impact of brucellosis on patients and their families. The goal is to understand the costs associated with diagnosing, treating, and managing brucellosis, as well as the financial burden it places on households. Your responses will help us estimate the total economic burden of brucellosis in our region and inform policies to reduce its impact. Your answers will be kept confidential and used only for research purposes. The data will be analyzed to calculate the direct medical costs (e.g., tests, medications, hospital stays), direct non-medical costs (e.g., transportation, meals), and indirect costs (e.g., lost income due to illness) associated with brucellosis. Your participation is voluntary, and you can skip any questions you are uncomfortable answering.

**How to Complete the Questionnaire:**

1. **Section 1: Demographic Information**
   - Provide basic details about yourself, such as age, gender, education level, and residence. This helps us understand the characteristics of brucellosis patients in our study.
2. **Section 2: Disease Information**
   - Answer questions about your brucellosis diagnosis, treatment, and any complications you may have experienced.
3. **Section 3: Direct Medical Costs**
   - Provide details about the costs of diagnostic tests, medications, hospitalization, surgeries, and rehabilitation services. If you don’t remember the exact costs, provide an estimate.
4. **Section 4: Direct Non-Medical Costs**
   - Share information about expenses such as transportation, accommodation, meals, and caregiver-related costs.
5. **Section 5: Indirect Costs**
   - Estimate any income lost due to missed work or premature mortality (if applicable).
6. **Section 6: Out-of-Pocket Expenses**
   - Provide the total amount you or your family paid for healthcare related to brucellosis.
7. **Section 7: Consent**
   - Confirm that you have provided accurate information to the best of your knowledge and provide your signature or thumbprint (if applicable).

All your responses will be kept confidential. Your name and personal details will not be shared, and the data will only be used for research purposes. We greatly appreciate your time and effort in completing this questionnaire. Your input is invaluable to our study and will contribute to improving healthcare policies for brucellosis patients.

**Section 1: Demographic Information**

Name (optional): ___________________________

Age: _________ year

Gender:

- - Male
  - Female

Marital Status:

- - Single
  - Married
  - Divorced/Widowed

Education Level:

- - Illiterate
  - Primary School
  - Secondary School
  - High School Diploma
  - University Degree

Occupation: ___________________________

Health Insurance Coverage:

- - Yes
  - No

Residence:

- - Urban
  - Rural

Supplemental Insurance

- - Yes
  - No

Insurance status

- - Rural insurance
  - Social Security
  - Armed forces
  - Iran health
  - No insurance
  - Others

**Section 2: Disease Information**

Date of Brucellosis Diagnosis: ___________________________

- Disease Stage:
  - Acute
  - Chronic
- Treatment Type:
  - Outpatient
  - Inpatient
- Complications (if any):
  - Osteomyelitis
  - Abscess
  - Joint Damage
  - Other (specify): ___________________________

**Section 3: Direct Medical Costs**

- Diagnostic Tests:
  - Rose Bengal Test: Cost _________
  - ELISA: Cost _________
  - Wright Test: Cost _________
  - Blood Culture: Cost _________
  - PCR Test: Cost _________
  - Imaging (X-ray, Ultrasound, CT Scan): Cost _________
- Medications:
  - Doxycycline: Cost _________
  - Rifampin: Cost _________
  - Streptomycin: Cost _________
  - Other (specify): ___________________________
- Hospitalization Costs:
  - Room Charges: Cost _________
  - Nursing Care: Cost _________
  - ICU Stay (if applicable): Cost _________
- Surgical Interventions:
  - Abscess Drainage: Cost _________
  - Joint Replacement: Cost _________
  17. Rehabilitation Services:
  - Physiotherapy: Cost _________

**Section 4: Direct Non-Medical Costs**

- Transportation Expenses: Cost _________
  Accommodation Costs: Cost _________
  Meal Expenses: Cost _________
  Caregiver-Related Costs:
  Time Off Work for Caregiver: Cost _________

**Section 5: Indirect Costs**

- Work Absenteeism (Days Missed): _________
  Estimated Lost Income Due to Absenteeism: Cost _________
  Premature Mortality (if applicable):
  - Age at Death: _________
  - Estimated Lost Income: Cost _________

**Section 6: Out-of-Pocket Expenses**

- Total Out-of-Pocket Healthcare Expenses: Cost _________

**Section 7: Consent**

I confirm that I have provided accurate information to the best of my knowledge.
- Signature (or thumbprint for illiterate participants): ___________________________
- Date: ___________________________

**Thank you**
